# Estimated intracranial volume from FreeSurfer is biased by total brain volume

**DOI:** 10.1186/s41747-018-0055-4

**Published:** 2018-09-19

**Authors:** Niklas Klasson, Erik Olsson, Carl Eckerström, Helge Malmgren, Anders Wallin

**Affiliations:** 1 Institute of Psychiatry and Neurochemistry, Department of Neuroscience and physiology, The Sahlgrenska Academy, University of Gothenburg, Wallinsgatan 6, 431 41 Mölndal, Sweden; 2 Institute of Medicine, Department of Internal medicine and clinical nutrition, The Sahlgrenska Academy, University of Gothenburg, Box 400, SE-405 30 Gothenburg, Sweden

**Keywords:** Bias, Brain, FreeSurfer, Intracranial volume, Magnetic resonance imaging

## Abstract

**Background:**

Estimated intracranial volume (eTIV) from FreeSurfer is not segmentation-based but calculated from the alignment of the input magnetic resonance (MR) images to the MNI305 brain atlas, an approach that could lead to a bias by total brain volume. If eTIV is unbiased, variance beyond that explained by intracranial volume should be random. Our null hypothesis was that no correlation would remain between eTIV and total brain volume when controlling for intracranial volume.

**Methods:**

eTIV and total brain volume for 62 participants were calculated on 1.5-T, T1-weighted MR images using FreeSurfer (version 6.0.0). Manual delineations of the intracranial volume were also made for the same images. To evaluate the null hypothesis, the partial correlation between eTIV and total brain volume was calculated when controlling for intracranial volume.

**Results:**

The partial correlation between eTIV and total brain volume when controlling for intracranial volume was 0.355 (*p* = 0.026). The null hypothesis was rejected.

**Conclusion:**

eTIV from FreeSurfer is biased by total brain volume.

**Electronic supplementary material:**

The online version of this article (10.1186/s41747-018-0055-4) contains supplementary material, which is available to authorized users.

## Key points

- eTIV from FreeSurfer is not calculated by delineation of the dura mater
- eTIV is biased by total brain volume
- eTIV might be suboptimal for intracranial volume normalisation

## Background

Intracranial volume is frequently estimated using the software suite FreeSurfer and is then commonly abbreviated as eTIV (estimated total intracranial volume) [1]. eTIV is automatically calculated by dividing a predetermined constant with the factor by which the input magnetic resonance (MR) images are scaled in size to align to the MNI305 head atlas [2–4]. The predetermined constant and the intracranial volume of the MR images are assumed to be similar to each other after the alignment, so division by the scaling factor should give a fair estimate of the intracranial volume before the alignment. The calculation of eTIV originates from a method described by Buckner et al. [5]. eTIV estimated by FreeSurfer has previously been reported to have Pearson correlations of 0.89–0.94 with manually estimated intracranial volume [6–8].

The alignment optimisation of the input MR images to the MNI305 brain atlas is done using Avi Snyder’s 4dfp suite of image registration tools [2, 9]. The resulting twelve-parameter affine transformation matrix tells the scaling, rotation, shearing, and translation of the aligned MR images in the three spatial dimensions, and its determinant gives the total factor by which the images were scaled during the alignment [3, 9, 10]. eTIV is then calculated by a predetermined constant (1948 mL) divided by the scaling factor [3, 11]. The predetermined constant is used to get a volumetric measure from the otherwise dimensionless scaling factor and was obtained as the slope of a linear regression without the intercept term, between manually estimated intracranial volume and corresponding scaling factors in 22 cases [3].

A potential problem of eTIV is that it is derived by the alignment of intensities not only in the skull, but also in the cerebrospinal fluid, fat, and brain tissue [12]. If a flawless alignment of brain tissues alone was done, the resulting scaling factor would be equal to the total brain volume of the atlas divided by the total brain volume of the MR images. Then, as both the predetermined constant and the brain volume of the atlas are constants, eTIV would become proportional to the total brain volume of the MR images and, therefore, depending on brain atrophy. The question is if the additional contribution of non-brain voxels to the alignment removes such a bias.

If eTIV is not biased, its variance should be random beyond that explained by the true intracranial volume. Our null hypothesis is that no correlation will remain between eTIV and total brain volume when controlling for fully delineated intracranial volume.

## Methods

Thirty-three memory clinic patients and 29 healthy controls were included from the Gothenburg MCI study [13, 14]. These participants had already been included in two previous studies about manual estimation of intracranial volume [15, 16]. The patients were classified as having either subjective cognitive impairment, mild cognitive impairment or probable dementia by the global deterioration scale for assessing cognitive impairment [14, 17]. Demographics are shown in Table 1.

**Table 1:** Demographics

Group	All participants	Healthy controls	Probable dementia
Number of participants	62	29	25
Gender (male/female)	23/39	8/21	11/14
Age (years)	66.1 ± 8.0	66.4 ± 7.5	65.5 ± 8.8
Education (years)	11.0 (6.0, 23.0)	11.5 (7.0, 15.0)	10.0 (6.0, 23.0)
MMSE	28.5 (16.0, 30.0)	30.0 (27.0, 30.0)	25.0 (16.0, 30.0)
ICV (mL)	1506 ± 154	1498 ± 144	1512 ± 172
eTIV (mL)	1566 ± 158	1563 ± 154	1566 ± 171
TBV (mL)	1118 ± 120	1116 ± 113	1110 ± 138
eTIV versus ICV (mL)	59 ± 44	65 ± 46	53 ± 42
eTIV versus ICV (%)	4.0 ± 3.1	4.4 ± 3.2	3.6 ± 2.9

Age, manually delineated intracranial volumes (*ICV*), FreeSurfer estimated intracranial volumes (*eTIV*), total brain volumes (*TBV*), and the difference between ICV and eTIV (*eTIV* versus *ICV*) written as millilitres (mL) and percent of ICV are all described with means and standard deviations, while education and Mini-Mental State Examination (*MMSE*) are described with medians with minimum and maximum values enclosed in brackets. Gender is given as the number of males and females. Descriptive statistics of the sample subgroups “Healthy controls” and “Probable dementia” are presented to allow for comparisons with samples from similar previous studies

For each participant, MR images acquired with a three-dimensional, T1-weighted, magnetisation-prepared, rapid gradient echo (inversion recovery/gradient recalled) sequence on a 1.5-T Symphony scanner (Siemens Healthineers, Erlangen, Germany) were available. The acquisition parameters were as follows: inversion time 820 ms; repetition time 1610 ms; echo time 2.38 ms; flip angle 15°; field of view 250 × 203 mm; matrix 512 × 416; acquisition pixel spacing 1.0 × 1.0, reconstruction pixel spacing 0.49 × 0.49 mm; slice thickness 1 mm; spacing between slices 1 mm (no interslice gap); receiver bandwidth 220 Hz/pixel; number of slices 192; acquisition time 1.7–2.4 min; body transmit coil type.

### Manually estimated intracranial volume

The manual estimates of intracranial volume were included from a previous study [15] where the same participants and MR images were used. In the previous study, the intracranial volumes were estimated by delineating all sagittal intracranial areas in the MR images following the protocol described by Eritaia et al. [18].

### FreeSurfer estimates

eTIV and total brain volumes were calculated using FreeSurfer version 6.0.0 run on a MacPro 3.1 with two quad-core Intel Xeon 2.8-GHz, 64-bit processors, 8 GB of RAM, and Mac OS X version 10.8.5 (Apple Inc., Cupertino, CA, USA). The brain volume estimation in FreeSurfer is an automatic classification of voxels into brain tissue labels, described by Fischl et al. [19].

Visual inspection of the alignments from which eTIV was calculated did not reveal any obvious errors, so no exclusion because of alignment errors was performed. Visual inspection of the brain volume segmentations revealed partial inclusion of dura mater, missed voxels of cortex, partial inclusion of sinuses, and, in some cases, partial inclusion of porous bone. We did correct for these errors following the guidelines [20]. The error checking and corrections were done blinded to participant age, gender, and cognitive status.

### Statistical analysis

The median partial correlation [21] between eTIV and total brain volume when controlling for intracranial volume was calculated using delete-two Jackknife resampling [22, 23]. The null hypothesis was then tested using the Jackknife replicate with the lowest partial correlation to assure that no pair of data points had an exceptionally large impact on the partial correlation. The alpha value was set to 0.05. By simulation (Additional file 1), the risk of a false positive finding in the current setting should be about 5% or less, assuming that the manual estimates of intracranial volume have a Pearson correlation of 0.99 or higher to actual intracranial volume. Further, Pearson correlations were calculated between intracranial volume, eTIV, and total brain volume. All statistics were performed in MATLAB version R2015b (Mathworks, Natick, MA, USA).

## Results

The Jackknife replicate with the lowest partial correlation between eTIV and total brain volume when controlling for intracranial volume showed a significant correlation of 0.290 (*p* = 0.026). The median partial correlation for all Jackknife replicates was 0.355. Thus, about 13% (≈ 0.355^2^ x 100) of the variance in eTIV that was not explained by intracranial volume was explained by total brain volume.

The Pearson correlation coefficient between intracranial volume and eTIV was 0.960 for all participants, 0.954 for the controls, and 0.971 for participants with probable dementia. The Pearson correlation coefficient between intracranial volume and total brain volume was 0.921 for all participants, 0.934 for the controls, and 0.919 for participants with probable dementia. The Pearson correlation coefficient between eTIV and total brain volume was 0.923 for all participants, 0.928 for the controls, and 0.921 for participants with probable dementia.

On average, eTIV overestimated the intracranial volume by 4% (Table 1).

## Discussion

Our study found a significant partial correlation between eTIV and total brain volume when controlling for intracranial volume, so the null hypothesis was rejected. This finding implies that eTIV is biased by total brain volume and reinforces the doubts about eTIV construct validity. Unfortunately, the strong collinearity between intracranial volume and total brain volume makes it difficult to determine the exact extent of the bias.

Two longitudinal studies have previously evaluated the possibility of a total-brain-volume-dependent bias in eTIV estimation. To assess the bias, both studies calculated the Pearson correlation coefficient between the change in total brain volume and the change in eTIV over time [7, 24]. One of these studies found a Pearson correlation coefficient of 0.515 (*p* = 0.05, *n* = 11) when using FreeSurfer version 3.0.2 in participants with frontal lobe dementia [24]. The other study did not see any tendency towards a significant Pearson correlation when using FreeSurfer version 5.1.0 in healthy elderly (*p* = 0.892, *r* = − 0.019, *n* = 53) [7]. The small number of participants in the first of these studies might have contributed to the non-significant finding. In the second study, an increase in both the manual estimates of intracranial volume and eTIV was seen between baseline and follow-up. According to Nordenskjöld et al. [7], these volume increases might have been the effect of a system upgrade of the MR scanner, which could have interfered with the results.

Besides the use of different study designs, an explanation to why a total-brain-volume-dependent bias was seen in the present study and indicated by Pengas et al. [24], but not by Nordenskjöld et al. [7], could be the use of different participant groups. The MNI305 atlas is based on healthy young adults and the atlas brain has fairly small lateral ventricles. With larger lateral ventricles in the images to analyse, the ventricular volume might act to increase the size of the atlas during the alignment, thus counteracting the effect of cortical atrophy on eTIV. Further, cortical atrophy that only widens the sulci will likely have less impact on the alignment with regard to a total-brain-volume-dependent bias, as parts of the outer border of the brain still lies close to the dura mater. Conversely, a cortical atrophy that locally or globally retracts the gyri in combination with less ventricular enlargement might result in the brain-volume-dependent-bias to become more apparent. This could explain why Pengas et al. [24] almost found a significant bias though only following 11 participants; all of them had frontal lobe dementia. It could also explain why only a small bias was found in the present study where both healthy elderly and patients with different dementia diseases were included, and could help explaining why no bias was detected by Nordenskjöld et al. [7].

The Pearson correlation between eTIV and intracranial volume is stronger in the present study (*r* = 0.96) than in previously published studies. The Pearson correlation coefficient between eTIV and manually estimated intracranial volume typically ranges between 0.89 and 0.94 [6–8]. The mean percentage error in eTIV in the dementia group in the present study is similar to that found by Malone et al. (+ 3.7%) [8], who evaluated eTIV estimated from 288 participants with probable Alzheimer’s disease. In the study by Nordenskjöld et al. [7], FreeSurfer also tended to overestimate intracranial volume, but in spite of the possible scanner drift, the average overestimation of eTIV decreased with age. According to the expected total-brain-volume-dependent bias in eTIV, eTIV should reduce with atrophy. This is also the case in the present study where the overestimation was smaller in the dementia group (+ 3.6%) compared to the control group (+ 4.4%).

In a study by Hansen et al. [25], normalisation by eTIV decreased the sample sizes needed to detect a volume difference in hippocampal volume between two hypothetical groups. Linear normalisation by eTIV even outperformed linear normalisation using more valid estimates. However, it seems to be assumed that the mean volume difference between the two groups will not be affected by normalisation. Under this assumption, the smallest sample sizes will be achieved using intracranial volume estimates that explain the most variance in the volume of interest. The additional variance that eTIV explains compared to the more valid estimates risks being variance due to the total-brain-volume-dependent bias, which in reality might reduce the mean volume difference between the two groups during normalisation, thus reducing the gain of the normalisation.

Voevodskaya et al. [26] have evaluated ratio and linear regression normalisation when using eTIV from FreeSurfer version 5.1.0. In their study, there was only a very slight advantage in classification performance between controls, participants with mild cognitive impairment, and patients with Alzheimer’s disease when using hippocampal volumes normalised by eTIV instead of raw hippocampal volumes. The combined impression from two studies by Westman et al. [27] and Zhou et al. [28] is that ratio normalisation with eTIV is not beneficial for multivariate classification of controls and patients with Alzheimer’s disease, and questionable for univariate classification models. The small benefit of eTIV normalisation in these three studies could be due to a number of reasons, such as: (1) the total-brain-volume-dependent bias in eTIV; (2) the choice of normalisation method; and (3) the reduced need of normalisation when comparing groups with large mean volume differences.

In studies where total brain volume loss is small, the bias in eTIV will be small too, but even then it is probably better to use manual estimates of intracranial volume or even the total brain volume estimate from FreeSurfer. Lehmann et al. [29] report a Pearson correlation coefficient of *r* ≥ 0.98 between manually estimated total brain volume and the total brain volume estimate from FreeSurfer, a correlation stronger than those reported between eTIV and manual estimates of intracranial volume [6–8]. Thus, for samples with small total brain volume loss, total brain volume should have a better chance to reduce variance explained by premorbid total brain volume than eTIV has.

When troubleshooting the output from the FreeSurfer analyses, it is recommended to inspect the atlas alignment and correct it if necessary. The instructions for manual correction of the atlas alignment states that: “The goal is to stretch, translate, and rotate your moveable volume so that the two brains look as similar as possible, at least along the key anatomical points (anterior/posterior commissures, the temporal lobes in the coronal plane, and the midline cut)” [10]. The two brains mentioned in the instruction are those from the input MR images and the atlas (the movable volume). Following these instructions, there is a risk that eTIV is made to depend even more on the brain, as the alignment of the intracranial cavity or the skull is not considered.

Besides FreeSurfer, other automatic approaches for intracranial volume estimation exist. While these methods produce estimates with correlations between 0.86 and 0.99 to manually estimated intracranial volumes [7, 8, 30, 31], they too need more thorough evaluation. For now, manual estimation by the delineation of the dura mater is the safest way to obtain valid intracranial volume estimates in T1-weighted MR imaging. Just by delineating two selected intracranial areas, estimates with Pearson correlation coefficients to fully delineated intracranial volumes above 0.99 may be achieved [16]. The delineation of the dura mater minimises the risk of bias by brain morphology or total brain volume.

**Fig. 1:**
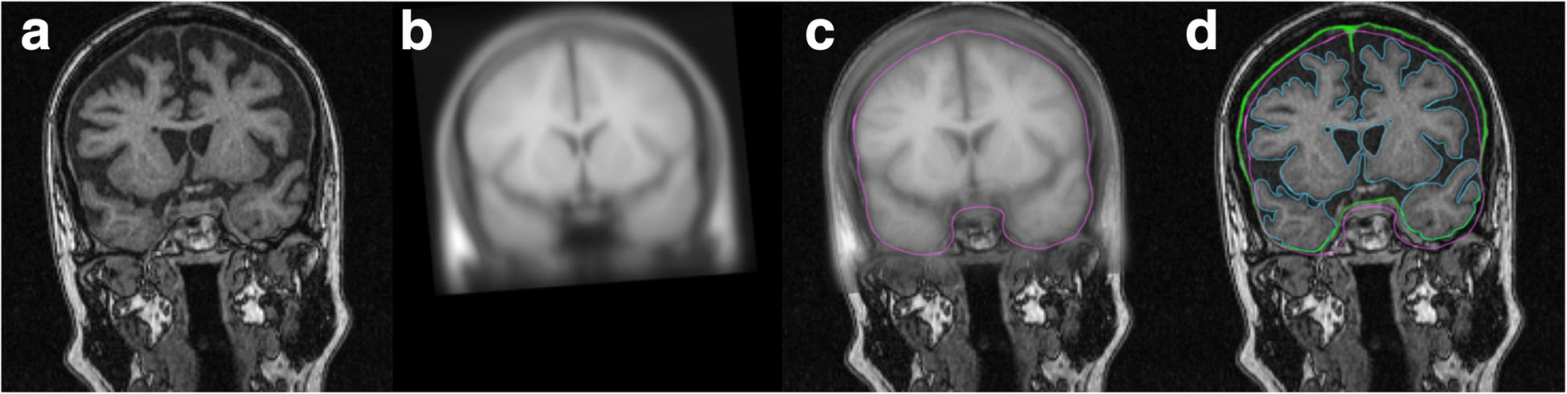
Illustration of FreeSurfer atlas alignment for estimation of intracranial volume. **a** T1-weighted image of a participant with frontal lobe dementia. **b** MNI305 atlas aligned to the T1-weighted image. **c** T1-weighted image with the aligned atlas overlaid and the intracranial surface of the atlas delineated (pink). **d** T1-weighted image with the brain surface (blue contour), the intracranial surface of the atlas (pink), and the actual intracranial surface (green) delineated; the atlas surface (pink) seems to follow an outer perimeter set by the gyri rather than following the intracranial surface (green)

The present study has limitations. Our interpretation of the rejected null hypothesis assumes that the manually delineated intracranial volume captures most of the variance of the actual intracranial volume. If this would not be true, the correlation between eTIV and total brain volume that is not explained by the intracranial volume estimates could be due to erroneously disregarded variance. However, the estimation approach behind eTIV, our use of fully delineated intracranial volume and the well-defined dura mater in the MR images (see Fig. 1) make such an explanation improbable. In addition, the exact extent of total-brain-volume-dependent bias in eTIV cannot be determined using the methodology of the present study and remains an issue for further investigations. Finally, we note that another issue for further investigation is how the total-brain-volume-dependent bias in eTIV varies with the use of MR scans with different acquisition parameters, field strengths, and scanner manufacturers.

In conclusion, we showed that eTIV from FreeSurfer is biased by total brain volume. Before more thorough evaluations or methodological improvements of eTIV become available, the use of eTIV in normalisation of regional brain volume should be considered with care.

## Additional file

Additional file 1: Simulation script that evaluates the delete-two Jackknife resampling approach that was used in the partial correlation analysis of the present study. The simulation evaluates the risk of getting a false positive finding if there is no total-brain-volume-dependent bias in estimated total intracranial volume from FreeSurfer. The different parameters in the MATLAB script may be changed to see how the risk of getting a false positive finding changes. The current parameters were set to resemble those of the present study. (M 12 kb)

## Abbreviations

eTIV: Estimated total intracranial volume from FreeSurfer
MR: Magnetic resonance
